# Supplementary material for: A Sex-Stratified Genome-Wide Association Study of Tuberculosis Using a Multi-Ethnic Genotyping Array
Source: Front Genet. 2019 Jan 18;9:678. doi: 10.3389/fgene.2018.00678 (PMC6346682; doi:10.3389/fgene.2018.00678)
Supplement: Supplementary file 1 [file Data_Sheet_1.docx]

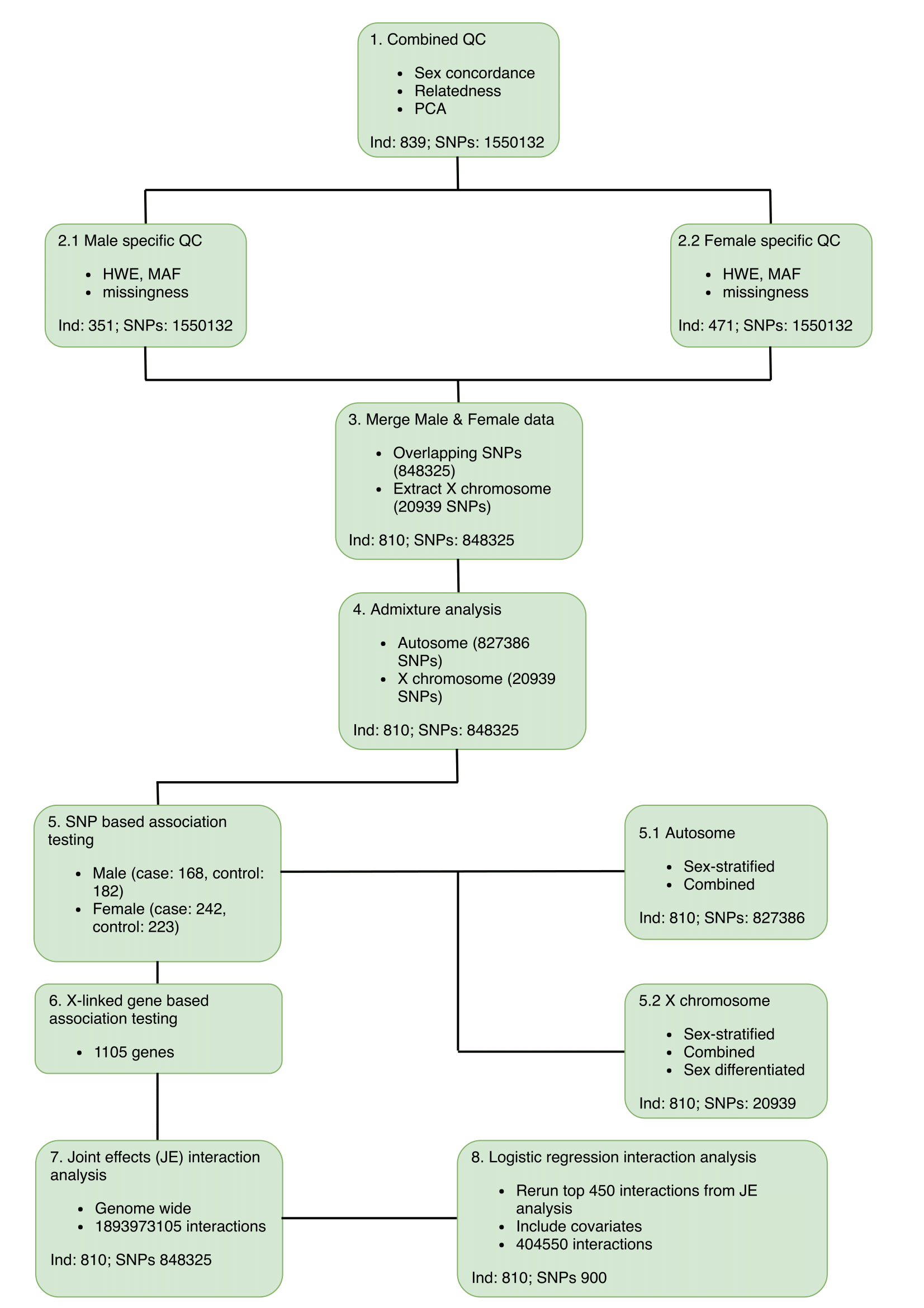


**Figure S1:** Flow diagram of data QC and association testing


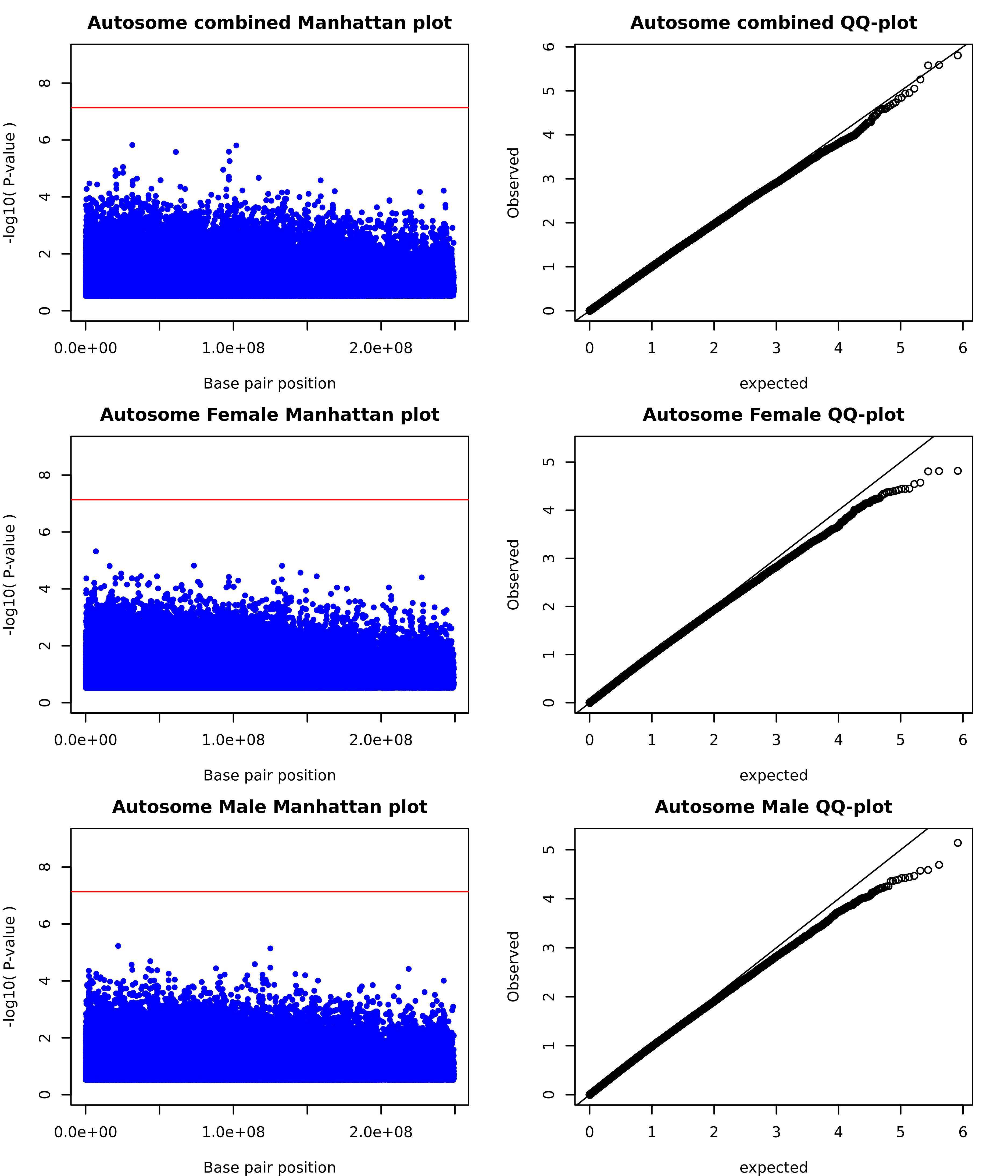


**Figure S2:** Manhattan plot and QQ plot for sex-stratified and combined analysis on the Autosome. Red line indicates significance threshold (5e^-8^).


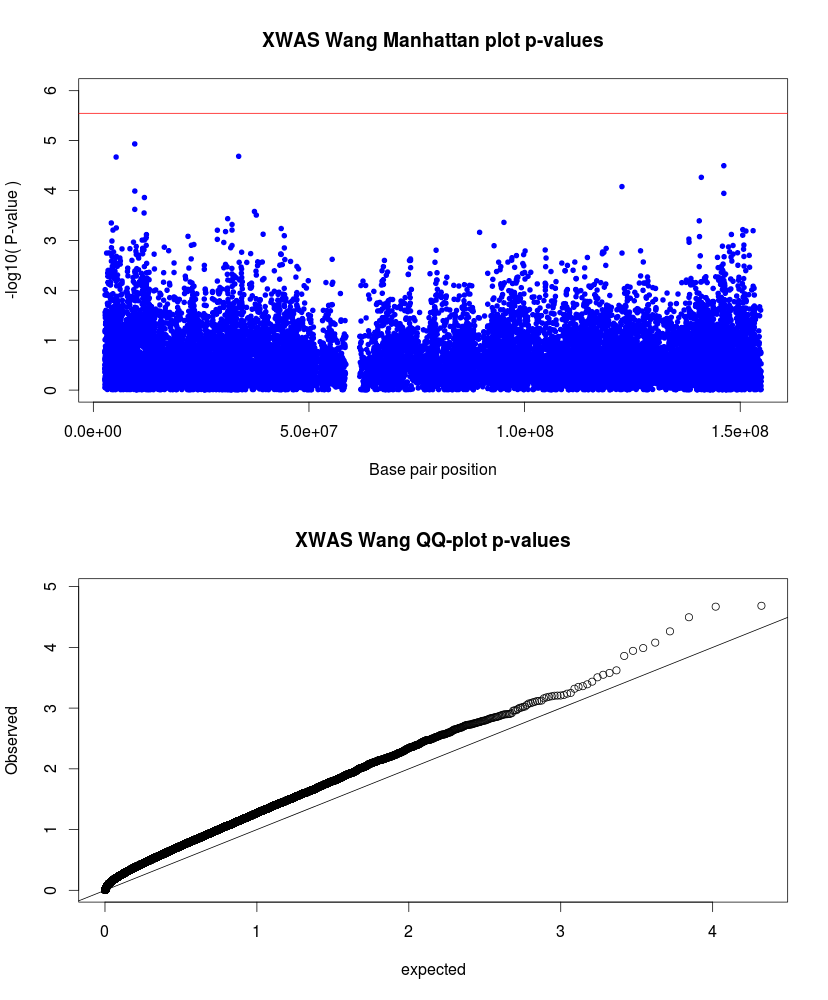


**Figure S3:** Manhattan and QQ-plot for X-linked SNP association testing including modelling for inactivation states, the red line indicates significance threshold of 2.8e^-6^. QQ-plot indicates inflated p-values and potential increase in type 1 errors.

**Table S1:** Most significant results for X-linked SNP association testing including modelling of X chromosome inactivation states.

| SNP | Gene | Location | Model | SE | LLR | OR | 95CI-L | 95CI-H | p | llrp |
| --- | --- | --- | --- | --- | --- | --- | --- | --- | --- | --- |
| rs768568 | TBL1X | Intron | Escape_of_XCI | 0.13 | 19.78 | 0.56 | 0.43 | 0.72 | 1.17e^-5^ | 8.69e^-6^ |
| rs6631824 | DMD | 5'UTR | Random_XCI | 0.09 | 18.69 | 0.69 | 0.59 | 0.82 | 2.07e^-5^ | 1.54e^-5^ |
| rs12011358 | NLGN4X | 3'UTR | Skewed_XCI_to_risk_allele | 0.10 | 18.33 | 1.51 | 1.25 | 1.82 | 2.14e^-5^ | 1.86e^-5^ |
| rs930631 | MIR506 | 3'UTR | Random_XCI | 0.08 | 17.42 | 1.41 | 1.20 | 1.65 | 3.19e^-5^ | 2.99e^-5^ |
| rs176024 | MAGEC3 | missense,utr variant 5 prime | Escape_of_XCI | 0.14 | 16.77 | 0.56 | 0.43 | 0.74 | 5.45e^-5^ | 4.22e^-5^ |
| X:9959944 | SHROOM2 | 5'UTR | Skewed_XCI_to_risk_allele | 0.49 | 16.28 | 0.21 | 0.07 | 0.49 | 1.32e^-3^ | 5.45e^-5^ |
| rs386827412 | GRIA3 | Intron | Skewed_XCI_to_risk_allele | 0.11 | 16.15 | 0.64 | 0.51 | 0.80 | 8.35e^-5^ | 5.86e^-5^ |
| rs17340554 | DMD | Intron | Skewed_XCI_to_risk_allele | 0.29 | 15.76 | 0.35 | 0.19 | 0.61 | 3.67e^-4^ | 7.17e^-5^ |
| rs5933749 | TBL1X | Intron | Escape_of_XCI | 0.14 | 15.54 | 1.72 | 1.31 | 2.27 | 1.03e^-4^ | 8.09e^-5^ |

**Table S2:** Results for genome wide interaction analysis using the joint effects model and no adjustment for covariates.

| **Chr1** | **SNP1** | **BP1** | **Gene1** | **Chr2** | **SNP2** | **BP2** | **Gene2** | **P-value** |
| --- | --- | --- | --- | --- | --- | --- | --- | --- |
| 23 | rs1823897 | 3082395 | ↑* ARSF | 23 | rs7064174 | 12359018 | FRMPD4 | 7.23e^-14^ |
| 23 | rs426247 | 31078534 | ↑ ARSE | 23 | rs68046754 | 91828549 | PCDH11X | 1.96e^-12^ |
| 5 | rs2112508 | 62761734 | LOC107986418 | 10 | rs1194709 | 54198592 | ↑ RPL31P44 | 2.81e^-12^ |
| 7 | rs1347075 | 13520443 | LOC107986770 | 8 | rs958374 | 9197661 | ↑ LOC157273 | 3.49e^-12^ |
| 23 | rs5991619 | 43146833 | ↓* MAOA | 23 | rs73535318 | 89712929 | RNU6-555P | 5.87e^-12^ |
| 13 | rs7991005 | 95034355 | GPC6 | 16 | rs2287072 | 55583550 | LPCAT2 | 5.95e^-12^ |
| 23 | Rs11094800 | 5611715 | ↓ NLGN4X | 23 | rs68046754 | 91828549 | PCDH11X | 7.20e^-12^ |
| 5 | rs7341174 | 117592341 | LINC02147 | 12 | rs1918191 | 79625762 | SYT1 | 7.63e^-12^ |
| 23 | rs68046754 | 91828549 | PCDH11X | 23 | rs6528958 | 141176547 | ↓ MAGEC2 | 8.37e^-12^ |
| 1 | rs6694239 | 175724222 | ↓ PAPPA2 | 2 | rs985256 | 201208692 | SPATS2L | 1.12e^-11^ |
| 23 | rs4824843 | 44476910 | ↑ FUNDC1 | 23 | rs58762927 | 91208477 | LOC107986770 | 1.29e^-11^ |
| 23 | rs2170314 | 97008577 | ↑ DIAPH2 | 23 | rs4633188 | 97033137 | ↑ DIAPH2 | 1.32e^-11^ |
| 5 | rs2112508 | 62761734 | LOC107986418 | 10 | rs1194716 | 54196773 | ↑ RPL31P44 | 1.33e^-11^ |
| 23 | rs5972637 | 32675036 | DMD | 23 | rs73535318 | 89712929 | RNY6-555P | 1.65e^-11^ |
| 23 | rs1352015 | 65843843 | EDA2R | 23 | rs68046754 | 91828549 | PCDH11X | 1.75e^-11^ |
| 23 | rs5916341 | 6135980 | NLGN4X | 23 | rs68046754 | 91828549 | PCDH11X | 2.50e^-11^ |
| 23 | rs3072699 | 6136515 | NLGN4X | 23 | rs68046754 | 91828549 | PCDH11X | 2.50e^-11^ |
| 23 | rs1823897 | 3082395 | ↑ ARSE | 23 | rs7053176 | 70849834 | ↑ CXCR3 | 2.59e^-11^ |
